# Neurofunctional correlates of a neurorehabilitation system based on eye movements in chronic stroke impairment levels: A pilot study

**DOI:** 10.1002/brb3.3049

**Published:** 2023-07-11

**Authors:** Bárbara R. García‐Ramos, Rebeca Villarroel, José L. González‐Mora, Consuelo Revert, Cristián Modroño

**Affiliations:** ^1^ Departamento de Ciencias Médicas Básicas Universidad de la Laguna Tenerife Spain; ^2^ Departamento de Medicina Física y Farmacología Universidad de la Laguna Tenerife Spain; ^3^ Instituto de Tecnologías Biomédicas Universidad de la Laguna Tenerife Spain; ^4^ Instituto Universitario de Neurociencia Universidad de la Laguna Tenerife Spain

**Keywords:** eye movements, fMRI, neurorehabilitation, stroke, virtual training

## Abstract

**Introduction:**

Rehabilitation after a stroke is widely considered fundamental to improve secondary functional impairments. Accessible methods based on motor learning, motor transfer and virtual environments are necessary to help to improve stroke patients’ quality of life.

**Objectives:**

Continuing the line of our previous studies, this work investigated the effect of our new and innovative game‐based virtual reality training using the control of virtual objects with gaze in three chronic stroke survivors.

**Methods:**

All participants performed an eye‐controlled virtual training task for 4 weeks. Pre‐ and post‐training evaluation were carried out with the Fugl‐Meyer Assessment for upper extremity scale as well as performing a tracking task inside an MRI scanner with a MRI‐compatible eye‐tracker or a joystick.

**Results:**

Neural results for each participant show the increase of activity in the motor cortex, basal ganglia and cerebellum for both effectors (hand or eye).

**Conclusion:**

These promising results have a potential application as a new game‐based neurorehabilitation approach to enhance the motor activity of stroke patients.

## Graphical abstract

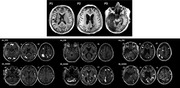

## 1 INTRODUCTION

Stroke is one of the most important and disabling brain disorders in the world today (Feigin et al., 2014). The Global Burden of Diseases, Injuries, and Risk Factors Study (GBD 2019 Stroke Collaborators, 2021) reports that the number of stroke related DALYs (disability‐adjusted life‐years) increased by 33.5 million from 91.5 million in 1990 to 125 million in 2019. Moreover, it has been predicted that the numbers of stroke survivors between 2015 and 2035 will increase by 34% in the European Union (Stevens et al., 2017).

Rehabilitation is the key to promoting improvements in stroke patients’ disabilities (WHO, 2021). Unfortunately, the lack of capacity of rehabilitation centers could lead to long delays in starting rehabilitation (Stevens et al., 2017) and most of the treatments need to use the affected limbs which is not always possible because of the patients’ limitations (Camporesi et al., 2013). It is, therefore, necessary to create new accessible methods for all stroke survivors.

In the field of neurorehabilitation, it is important to find proper strategies to increase cerebral activity that could promote a better functional recovery (Crofts et al., 2020). Considering this principle, it is necessary to bear in mind that it would be necessary to use motor learning mechanisms to promote neuroplasticity or motor transfer, which are fundamental concepts in neurorehabilitation. Neuroplasticity consists of reorganizing and structuring brain function in response to injuries and stimuli (Su & Xu, 2020) and has been considered useful in the rehabilitation of stroke survivors and is highly dependent on motor learning (Dayan & Cohen, 2011; Dimyan & Cohen, 2011; Small et al., 2013). Furthermore, motor transfer, which is based on the application of a learned skill in a different context or task, is one of the major outcomes of motor learning (Censor, 2013). On the other hand, the use of virtual reality can provide training opportunities for many stroke patients to improve their adherence to treatment (Brunner et al., 2016; Krakauer, 2006), through more repetitive and engaging training that could also enhance neuroplasticity (Kim et al., 2020).

Based on the issues mentioned above, an approach to neurorehabilitation has been developed by the authors based on training eye control of virtual objects, which has been tested on healthy volunteers (García‐Ramos et al., 2021; Gebert et al., 2017; Modroño et al., 2020; Modroño et al., 2015; Modroño et al., 2013). The objective of this study is to explore the effect of this new game‐based ocular virtual reality training in three different profiles of stroke survivors.

Based on the results obtained in healthy volunteers, it can be hypothesized that: (a) virtual training with ocular movements in stroke participants should lead to an increase of cerebral activity in sensorimotor regions that (b) can be associated with an increase in the accuracy in eye movements, which can be transferred to the hand. This virtual training could be a useful tool in neurorehabilitation to increase cerebral activity by activating motor systems to help to improve recovery in stroke survivors.

## 2 METHODS

### 2.1 Participants

Three chronic stroke survivors (1 female, 2 males) with different levels of impairment were recruited from the Association for Acquired Brain Injury of Tenerife (ADACEA‐TF), to participate in the experiment. All participants suffered from ischemic or hemorrhagic stroke and had normal or corrected to normal vision. The experimental study was approved by the local Ethics Committee (University of La Laguna; registry number: CEIBA2015‐0178; The Ethics Committee for Drug Research of the Hospital Complex University of the Canary Islands CHUC_2020_48), and written informed consent was obtained from all participants in accordance with the Declaration of Helsinki.

### 2.2 Motor and functional assessment

An initial evaluation was carried out to check the baseline of participants in the pretraining (Table 1). In order to do this, the researchers valued spasticity using the Modified Ashworth Scale (Bohannon & Smith, 1987), degree of dependence in daily activities assessed by the Modified Rankin Scale (van Swieten et al., 1988), the capacity to execute basic everyday activities with the Spanish version of the Barthel Index (Baztán et al., 1993), grade of impairment caused by the stroke by the adapted version in Spanish of the National Institutes of Health Stroke Scale (NIHSS) (Domínguez, 2006), and evaluated conscious level by the Glasgow Coma Scale (Teasdale & Jennett, 1974). On the other hand, upper limb sensorimotor functioning was assessed before and after intervention by a physiotherapist with the Fugl‐Meyer Assessment for upper extremity scale (FMA‐UE) to evaluate motor recovery. The motor domain for the upper limb has a maximum score of 66 points (Fugl‐Meyer et al., 1975). This scale is highly recommended for clinical use in stroke research (Sullivan et al., 2013).

**TABLE 1:** Baseline measurements.

Participant	Age	Stroke type	Sex	Side of lesion	MAS	MRS	BI	NIHSS	GCS	Level of impairment
P1	61	I	M	L	0	0	100	1	15	Low
P2	63	H	M	R	2	4	80	3	15	Medium
P3	78	I	F	L	1+	4	20	16	7	High

H, hemorrhagic; I, ischemic; M, male; F, female; L, left; R, right; MAS, Modified Asworth Scale; MRS, Modified Ranking Scale; BI, Barthel Index; NIHSS, National Institutes of Health Stroke Scale; GSC, Glasgow Coma Scale; P1, participant 1; P2, participant 2; P3, participant 3.

**FIGURE 1:**
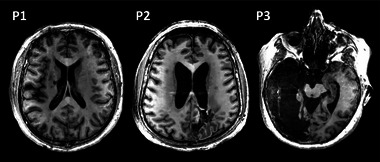
Axial anatomical images of brain damage in each participant. P1, participant 1; P2, participant 2; P3, participant 3.

**Case 1**:

The first participant was a 61‐year‐old male, who had suffered an ischemic stroke of the left anterior circulation 13 months prior to the experiment (Figure 1). He had a total degree of independence and capacity to execute basic activities in daily life. There was no presence of spasticity in the affected limbs and he had a maximum level of consciousness. In addition, the NIHSS showed a low degree of deterioration caused by the stroke. After the initial evaluation based on the motor and functional assessment scales, P1 (participant 1) was classified as having a low level of impairment.

**Case 2**:

The second participant was a 63‐year‐old male, who had suffered a hemorrhagic stroke of the right hemisphere in the occipital‐parietal area 10 months before the experiment (Figure 1). He had difficulties performing some of his bodily needs. However, he had a low‐level degree of dependency in the Barthel Index. He presented a marked increase in muscle tone across the whole range of motion, a maximum level of consciousness and a slight deterioration caused by the stroke according to the NIHSS. After the initial evaluation based on the motor and functional assessment scales, P2 (participant 2) was classified with a medium level of impairment.

**Case 3**:

The third participant was a 78‐year‐old female, who had suffered an ischemic stroke of the left hemisphere in the occipital‐parietal area 49 months prior to the experiment (Figure 1). She was unable to attend to her bodily needs and to walk without assistance, which correlate with a severe degree of dependency according to her score in the Barthel Index. She presented a slight increase in tone during passive movements, no motor response and difficulties to communicate due to the use of inappropriate words. Moreover, she showed a severe degree of deterioration as measured by NIHSS. After the initial evaluation based on the motor and functional assessment scales, P3 (participant 3) was classified with a high level of impairment in this research.

### 2.3 Intervention task

**FIGURE 2:**
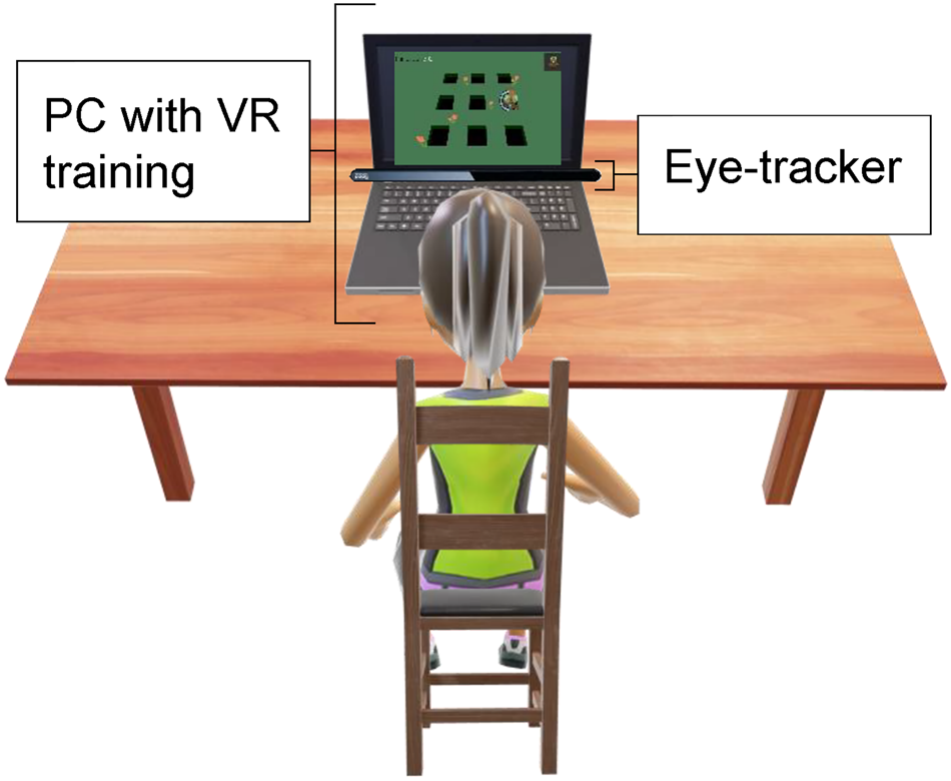
Experimental environment where the participant practices with an eye‐tracker controlled by ocular movements in a PC with a virtual task.

The intervention was based on a neurorehabilitation system composed by a laptop, an eye‐tracker device (Tobii 4c—https://gaming.tobii.com) and arcade games in a virtual environment. The games were developed in Unity game engine (Unity technologies, San Francisco, CA, United states) used under Windows OS (Figure 2).

The software involved four tasks inspired by classic arcade games such as memory, whac‐a‐mole, adventure game and target shooting controlled by eye‐tracking (the use of the upper limbs was not necessary). When the player starts the game, they select their name and freely choose the game they want to play with their gaze. All games are structured by increasing levels of difficulty with a scoring system, performance, visual and auditory feedback and instructions. Also, some of them have a chronometer to measure the total time and/or countdown tasks to improve the motivation of the participant with and without limitations of time. The game performance data (date, time employed, game selection) was registered to confirm that all the participants had played the games as instructed. First, in the memory game the participants have to match pairs of cards by moving a virtual cursor with their eyes. In the whac‐a‐mole game, the participants have to fix their gaze on the animal that appears in a hole on the screen. Adventure games are one the most complex games due to the character movements you need to practice your visual precision to get to the shelter and dodge obstacles. Finally, target shooting implies more capacity of reaction and velocity since targets appear suddenly on the screen in different zones where the participants have to shoot.

The neurorehabilitation system was installed in the participants’ homes, and both the participants and their caregivers were trained about the calibration of the eye‐tracker and game mechanisms, as access to the game, profile and game selection. The participants underwent a 4‐week intervention with virtual training that consisted of several video games. They had to practice daily for a period of 20–30 minutes, which could be at different times of the day. The researchers kept in touch once a week with the participants by phone or in person during the training period for better follow‐up.

### 2.4 fMRI scanning and MR image acquisition

**FIGURE 3:**
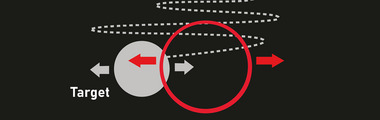
Continuous tracking task performed during fMRI in the pre‐ and posttraining evaluation. All participants used the eye as an effector in the first run and the hand in the second run.

During the fMRI scanning, which was performed on two different days (assessments) (pretraining evaluation/posttraining evaluation), participants executed a continuous tracking of a target (Figure 3) using a joystick (Resonance Technology, Inc., Northridge, CA) or their gaze depending on the run. Visual stimuli were delivered via MRI‐compatible eye‐glasses (Resonance Technology, Inc., Northridge, CA) in each run. No audio stimuli were given. The participants were asked to move their head as little as possible during the experiment. Each day involved two fMRI runs. During the first run, all the participants performed the task using their gaze with the MRI‐compatible eye‐tracker. The second run was then performed by using their healthy or affected hand depending on the state of the paretic arm (only one participant (P3) was not able to use the affected hand because of limitations in the paretic arm and was included in the second run playing with the nonparetic hand). The fMRI‐tracking task was the same for the pretraining evaluation and the posttraining evaluation. Before the intervention they had a 5‐min practice session to familiarize themselves with the motor task. Logs during the fMRI were recorded in all participants for behavioral analysis, and the mean absolute error (MAE) was used to evaluate motor performance. MAE is the most commonly used measure of overall accuracy in tracking tasks, and indicates the mean average distance the tracking circle was away from the target irrespective of the side (Jones, 2015).

A 3T Signa HD MR scanner (GE Healthcare, Waukesha, WI) was used to obtain axially oriented functional images using an echo‐planar‐imaging gradient‐echo sequence, an 8‐channel head coil (TR = 2000 ms, TE = 22 ms, flip angle = 75°, matrix size = 64 × 64 pixels, 36 slices, 4 × 4 mm in plane resolution, spacing between slices = 4 mm, slice thickness = 3.3 mm, interleaved acquisition). Alignment of the slices was performed to the anterior commissure‐posterior commissure line and covered the whole brain. Moreover, high‐resolution sagittally oriented anatomical images were collected for anatomical reference. A total of 2 (days) × 2 (runs) = 4 fMRI runs were performed. The fMRI run consisted of 10 tracking blocks of 20 s, separated by 20 s fixation blocks. A total of 210 volumes were taken for every participant in each run.

### 2.5 fMRI task

The fMRI task consisted of the continuous tracking of a target moving horizontally in a sine‐cosine waveform (Figure 3) in which the participant must try to pursue the target as closely as possible by using a sensor. A red circle (cursor) appeared on a black screen to track the gray circle. The experimental task had two conditions: *fixation* (participant must focus their gaze on a gray cross: that is, a basal condition) and *tracking* (tracking blocks). Custom software using Visual C# and DirectX was developed to implement the task. The same task was used and described in a previous neuroimaging study (Modroño et al., 2020).

### 2.6 Image preprocessing and analysis

fMRI data processing was performed using SPM12 software (www.fil.ion.ucl.ac.uk/spm/). In the preprocessing step the functional images were spatially realigned, unwarped, normalized, and smoothed. Visual inspection was used to validate normalized images. The normalized images of 2 × 2 × 2 mm were smoothed by a full width at half maximum (FWHM) 8 × 8 × 8 Gaussian Kernel. To model the BOLD response in each experimental condition, a block design in the context of a general linear model was implemented for individual subject analyses. Two SPM models were used here: one model for the eye‐controlled task and the other for the hand‐controlled task. For each model, the design matrix included two sessions (pretraining and posttraining evaluation) with two conditions each (tracking and fixation). The conditions were modeled using a box‐car function convolved with the hemodynamic response function (HRF). Activation maps were generated with two contrasts of interest: *eye‐tracking‐post > eye‐tracking‐pre* and *hand‐tracking‐post > hand‐tracking‐pre*. Statistical maps were generated for each participant by applying t statistics and were set at a voxel‐level threshold of *p* < .05, FDR corrected, and a minimum cluster size (*k*) of 10 voxels.

## 3 RESULTS

### 3.1 Behavioral results

**FIGURE 4:**
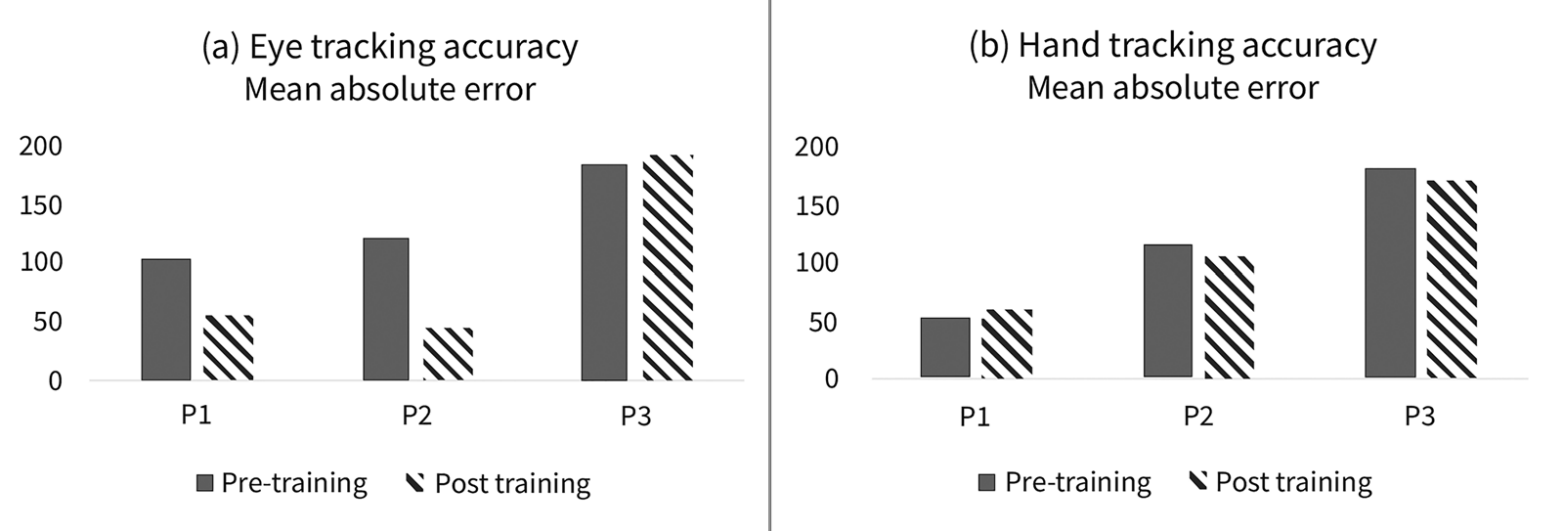
(a) Eye‐tracking accuracy during the posttraining evaluation for three stroke participants. (b) Hand‐tracking accuracy during the posttraining evaluation for three stroke participants.

The FMA scale for the upper extremity (FMA‐UE) showed an improvement of two points (pre: 59, post: 61) in P2 after 4 weeks’ intervention. There were no changes in the total scores of the other two participants. P1 obtained the maximum score (pre: 66, post: 66) and P3 a low score (pre: 8, post: 8) in both assessment periods. As regards the variations of the mean absolute error (MAE) between the fMRI pre‐ and posttest for the task performed with the eye, the MAE decreased 50.18 points in P1 and 78.90 points in P2, and increased 5.14 points in P3. For the task performed with the hand, the MAE decreased 10.31 points in P2 and 13.60 points in P3, and increased 7 points in P1 (Figure 4). Thus, these results show more accuracy in P1 and P2 (but not in P3) concerning eye‐tracking and a slightly better accuracy for P2 and P3 (but not for P1) in hand‐tracking.

### 3.2 Neural results

**FIGURE 5:**
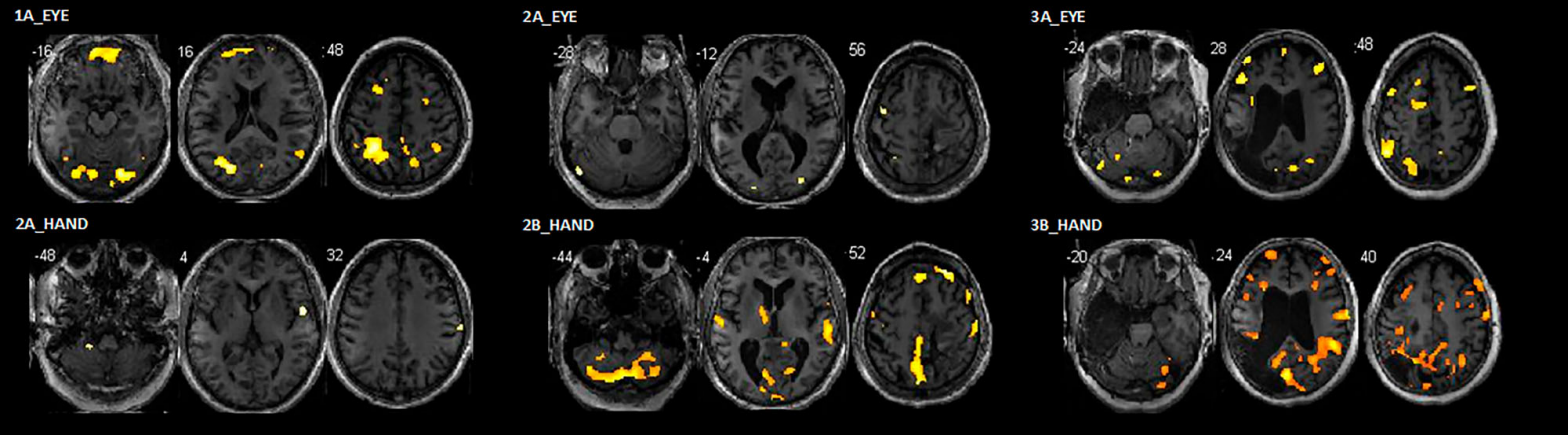
The increases of neural activations in participants **1**, **2,** and **3** after the virtual training period. (**a**: In the first run, participants performed the tracking task with the eye. **b**: In the second run, participants performed the tracking task with the hand). Threshold: *p* < .05. FDR corrected at the voxel level. (Contrasts: *eye‐tracking‐post > eye‐tracking‐pre* and *hand‐tracking‐post > hand‐tracking‐pre*).

The neural results of the two contrasts (*eye‐tracking‐post > eye‐tracking‐pre* and *hand‐tracking‐post > hand‐tracking‐pre*) reveal an increase of activations in cortical and noncortical regions in the three participants for both runs using their gaze or the joystick, including motor areas (Tables 2 and 3). Three representative slices were selected for each participant in order to show some of the activated brain regions (Figure 5).

**TABLE 2:** Main anatomical structures associated with motor activity and activated by the effect of ocular virtual training in the run performed with the eye (Contrast: *eye‐tracking‐post > eye‐tracking‐pre*).

**P1**				
**Region**	**BA**	**Cluster size (voxels)**	**[*X Y X*]**	**Peak T value**
Left cerebellum		56	–32 –58 –36	3.73
Left cingulate gyrus	5,7	273	–8 –48 28	3.94
Left inferior parietal lobe	7	1015	–30 –50 48	5.56
Left precentral gyrus	6	28	–36 –4 38	3.66
Left superior and medial frontal gyrus	8	23	–12 30 44	3.49
Right precuneus	7	78	12 –64 48	4.01
Left middle frontal gyrus	6,8	69	–26 14 50	4.07
Right middle frontal gyrus	6	72	28 0 52	4.06
Right inferior parietal lobe and postcentral gyrus	5,7	94	20 ‐50 56	3.71
Left cerebrum precentral gyrus	6	57	‐14 ‐20 68	3.38
Left superior frontal gyrus and supplementary motor area	6	10	‐8 10 58	3.19

**P2**				
**Region**	**BA**	**Cluster size (voxels)**	**[*X Y X*]**	**Peak T value**
Left cerebellum		30	–50 –72 –28	5.60
Left precentral gyrus	6,9	32	–50 0 38	4.82
Left superior and inferior parietal lobe	7	31	–28 –56 52	4.94
Left precentral gyrus	6	26	–42 –6 56	5.15

**P3**				
**Region**	**BA**	**Cluster size (voxels)**	**[*X Y X*]**	**Peak T value**
Right cerebellum		70	8 –64 –32	3.45
Left cerebellum		43	–40 –70 –22	3.78
Right cerebellum		45	22 –80 –20	3.45
Left insula	13	149	–42 –16 16	4.00
Left insula and putamen	13	33	–28 –12 14	4.03
Right insula	13	89	46 –38 20	3.45
Left middle and inferior frontal gyrus	6,8	661	–44 8 40	4.86
Inferior parietal gyrus	7	242	36 –52 36	3.71
Right middle frontal gyrus	8	180	38 12 44	3.73
Left inferior parietal lobule	7	889	–46 –40 54	4.60
Left supplementary motor area	6	144	–16 –2 42	3.74
Left supplementary motor area		18	–14 18 48	2.86
Right middle frontal gyrus	6	29	30 –2 56	2.94
Right superior frontal gyrus and right/left supplementary motor area	6	80	8 8 62	3.20
Left superior frontal gyrus and supplementary motor area	6	13	–12 6 62	3.04

The MNI coordinates of peak activity are represented in each cluster. Threshold *p* < .05. FDR corrected al the voxel level.

**TABLE 3:** Main anatomical structures associated with motor activity and activated by the effect of ocular virtual training in the run performed with the hand (Contrast: *hand‐tracking‐post > hand‐tracking‐pre*).

**P1**				
**Region**	**BA**	**Cluster size (voxels)**	**[*X Y X*]**	**Peak T value**
Left cerebellum		14	–20 –42 –48	4.61
Right superior temporal and precentral gyrus	6	41	54 –2 4	5.33

**P2**				
**Region**	**BA**	**Cluster size (voxels)**	**[*X Y X*]**	**Peak T value**
Left and right cerebellum		5819	–16 –80 –14	6.16
Left putamen		189	–10 –12 6	4.09
Left superior parietal lobe	7	66	–24 –82 32	4.23
Right middle and inferior frontal gyrus	6,8	234	50 16 38	5.29
Right superior frontal gyrus	8	286	24 38 52	5.96
Left paracentral lobe, superior parietal gyrus and supplementary motor area	4, 5, 6, 7	1308	–2 –32 50	5.29
Right postcentral gyrus	6	110	62 –16 50	4.23
Left precentral gyrus	4	26	–44 –18 48	3.65
Left cerebrum superior frontal gyrus and supplementary motor area	8	90	–4 30 52	4.86
Left precentral gyrus	4,6	12	–54 –6 52	3.22
Left superior frontal gyrus and right/left supplementary motor area	6,8	31	0 10 56	3.62
Superior and middle frontal gyrus	6,8	177	38 4 62	4.93
Left precentral gyrus	4	17	–28 –32 60	3.10
Left precentral and postcentral gyrus	4,6	37	–44 –14 62	4.15
Left superior frontal and precentral gyrus	6	107	–30 –10 68	4.17
Right superior frontal gyrus and supplementary motor area	6	98	14 6 66	4.91

**P3**				
**Region**	**BA**	**Cluster size (voxels)**	**[*X Y X*]**	**Peak T value**
Left paracentral lobule, superior and inferior parietal lobe, middle temporal gyrus and left supplementary motor area	4, 5, 6, 13	9075	–4 –46 62	6.90
Left cerebellum		81	–16 –80 –32	4.66
Right middle frontal gyrus	6,8	1200	40 18 34	4.42
Insula	13	266	–44 24 6	4.11
Right putamen		67	24 –8 14	3.36
Right postcentral and precentral gyrus	4,6	569	62 –8 22	5.88
Left precentral gyrus	6	20	–38 –6 32	2.95
Right middle frontal gyrus	6	20	28 –2 46	2.88
Right supplementary motor area	6	15	8 0 50	2.85
Left medial frontal gyrus and left/right Supplementary motor area	6	50	–2 –10 56	4.07
Right middle frontal gyrus	6	67	28 –10 58	4.38
Right superior and middle frontal gyrus	6,8	43	18 32 58	3.37
Right precentral and postcentral gyrus	4	15	40 –28 62	3.08

MNI coordinates of peak activity are represented in each cluster. Threshold *p* < .05. FDR corrected al the voxel level.

## 4 DISCUSSION

The present study explores the effect of the authors’ innovative neurorehabilitation approach based on the use of gaze to control virtual objects in three chronic stroke participants. It was hypothesized that (a) virtual training with ocular movements in stroke participants should lead to an increase of cerebral activity in sensorimotor regions that (b) can be associated with an increase in the accuracy in eye movements, which can be transferred to the hand.

In the FMA‐UE scale, one participant (P2) increased the total score by two points after the training period. This change took place in the subsection of coordination and speed. The other two participants had the same total score as in the pretraining evaluation, which was to be expected since P1 obtained the maximum score in the pretraining evaluation and P3 was not able to move her arm. It should be noted that in the context of the MCID (Minimum Clinically Important Difference), the increase of the score of P2 for the FMA‐UE did not achieve a change equal or superior to four points to be considered clinically relevant (Page et al., 2012; Lundquist & Maribo, 2016), unlike other studies on similar neurorehabilitation systems, which were based, for example, on virtual reality and planar motion exercises (Park et al., 2019), arm support and rehabilitation games (Prange et al., 2015), or robot‐assisted rehabilitation (Ranzani et al., 2020). The differences in clinical relevance between such works and ours can be due to the sample size, and, for this reason, it would be necessary to perform new studies with more participants to clarify the clinical effect of our virtual training approach on the FMA‐UE scale.

After the practice with the eye‐controlled training games task, the accuracy was increased (that is, the MAE decreased, Figure 4a) for the eye‐controlled fMRI task in two of the three participants (meanwhile a slight accuracy decrease was found for the other one). As regards the hand‐controlled fMRI task, the accuracy was slightly increased (Figure 4b) also for two of the three participants (meanwhile the accuracy was slightly decreased for another participant). The most noteworthy behavioral results are those obtained for participant 2, who had a middle level of impairment, and showed improvements in all the assessments (FMA‐UE and MAE for eye and hand‐controlled modalities). Taking together, these results seem to indicate that the practice with the eye‐controlled training games could have led to a better performance in the eye‐controlled sensorimotor task (that is, intertask transfer of motor learning) and, to a lesser extent, in the hand‐controlled task (that is, intertask and intereffector transfer of motor learning). Here it should be noted that this is an exploratory study, and caution must be taken when making interpretations; however, the present results appear to be partially consistent with other studies in which the participants used the hand as an effector in a sensorimotor training task and obtained improvements (Ewolds et al., 2017; Lang et al., 2013). Furthermore, this study is a step forward to continuing the line of our previous research with healthy volunteers where improvements in hand‐tracking accuracy were observed after a period of training in an eye‐controlled task (Modroño et al., 2020).

The neural results of the three chronic stroke participants show an increase of activity in sensorimotor regions, which is also consistent with our line of previous experiments in healthy volunteers (García‐Ramos et al., 2021; Gebert et al., 2017; Modroño et al., 2020; Modroño et al., 2015; Modroño et al., 2013). During the fMRI task, the contrasts (*eye‐tracking‐post > eye‐tracking‐pre* and *hand‐tracking‐post > hand‐tracking‐pre*) in the eye‐controlled and in the hand‐controlled runs show neural activity increases after the training period, particularly in the premotor cortex, primary motor area, cerebellum, basal ganglia, putamen, and insula. This is also consistent with other fMRI research works where the use of virtual reality training or physical therapy was found to increase brain activity in similar regions (Ackerley et al., 2016; Hardwick et al., 2015; Mekbib et al., 2021; Vourvopoulos et al., 2019; Xiao et al., 2017).

The premotor cortex and the primary motor area are associated with motor control (Kantak et al., 2012; Ohbayashi, 2021; Vourvopoulos et al., 2019) and are strongly related to motor learning (Doyon & Benali, 2005). It should be mentioned here that some participants presented activations in the SMA (supplementary motor area), which has been related with different aspects of motor learning (Ruan et al., 2018), including intereffector motor transfer (Jung et al., 2019; Modroño et al., 2020; Perez et al., 2008; Perez et al., 2007) that could be affected in participants with motor disabilities such as stroke (Dirren et al., 2021). The most extended activations in this type of experiment appeared in the cerebellum, typically involved in the learning of sensorimotor tasks (Hardwick et al., 2013) and related with intermanual transfer tasks (Obayashi, 2004). Furthermore, the increase of activity in basal ganglia is associated with sensory motor training that could support casual changes in neuroplasticity in chronic stroke survivors (Zastron et al., 2019). The putamen and insula, in particular, play an important role in planning, executing, and learning motor skills (Doyon et al., 2009). In sum, an increase of activity in brain areas related to motor aspects with only the training on ocular movements in a virtual environment was observed in the present experiment.

The differences observed in the results might be due to the heterogeneity of the lesions of the participants. It should be noted that P1 was classified with a low level of impairment with a left ischemic stroke, P2 a medium level with a right hemorrhagic stroke and P3 a high level with a left ischemic stroke. The idea that motor recovery is a multifactorial process, which depends on multiple clinical factors such as lesion type, topography, size, and the baseline of somatosensory integrity after the stroke, is supported by other research works (Feydy et al., 2002; Ingemanson et al., 2019). Many factors could influence the recovery process for motor impairments; for example, the site of occlusion in the posterior circulation is associated with worse outcomes than in the anterior circulation (Sommer et al., 2018), the low baseline in NIHSS score is a predictor of good clinical results (Alexandre et al., 2021), or the kind of exercises used in rehabilitation, such as core exercises that are effective for improving motor recovery in stroke patients with severe motor impairments (Bacho & Khin, 2023).

The present study has some limitations. First, as a pilot study, the sample size is small which does not allow for the generalizability of the results. However, it is a valuable first approximation that allows for the assessment of the potential of this new neurorehabilitation tool. Second, the sample is heterogeneous concerning to the type of stroke and other clinical factors, which makes it difficult to compare the results of the participants. Nevertheless, it also may offer a unique perspective that can be built upon in future research. It should be noted that the study is a proof of concept, and further research with larger sample sizes and a control group is needed to confirm these first preliminary promising findings.

## 5 CONCLUSIONS

To the best of the authors’ knowledge, this is the first time that stroke participants have performed an eye‐controlled neurorehabilitation task. The present work shows that training on the ocular control of virtual objects can be a useful tool to increase neural activity in motor areas of interest that may be useful for neurorehabilitation in clinical practice and generate mechanisms to promote motor learning or motor transfer. This work expands our previous studies with healthy participants. The activations results obtained in the motor cortex, basal ganglia, and cerebellum of the three participants suggest that this new game‐based neurorehabilitation approach has a promising potential application to help different types of stroke patients without resorting to limb movements. Therefore, it also invites us to continue this line of research by expanding the sample size.

## AUTHOR CONTRIBUTIONS

Conceived and designed the experiments: BRGR, RV, JLGM, CR, CM. Performed the experiments: BRGR, RV, CM. Analyzed the data: BRGR, RV, CM. Contributed reagents/materials/analysis tools: BRGR, RV, JLGM, CR, CM. Wrote the paper: BRGR, RV, CM.

## CONFLICT OF INTEREST STATEMENT

The authors declare that they have no conflict of interest.

### CONSENT TO PARTICIPATE

A written informed consent was obtained from all participants in accordance with the Declaration of Helsinki.

### PEER REVIEW

The peer review history for this article is available at https://publons.com/publon/10.1002/brb3.3049.

## Data Availability

The data that support the findings of this study are available from the corresponding author upon request.
